# Anal Papilloma: An Exceptional Presentation of Fibrocystic Disease in Anogenital Mammary-Like Glands

**DOI:** 10.1155/2015/426835

**Published:** 2015-10-01

**Authors:** Priya Subashchandrabose, Muthuvel Esakkai, Palani Venugopal, Ilavarasan Kannaiyan, Chitra Srinivasan, Punuru Tejashwini Reddy, Evelyn Elizabeth Ebenezer

**Affiliations:** ^1^Department of Pathology, Saveetha Medical College, Chennai 602105, India; ^2^Department of Surgery, Saveetha Medical College, Chennai 602105, India

## Abstract

Previously ectopic breast tissue was thought to be derived from the caudal remnants of the primitive embryonic milk ridges; anogenital mammary-like glands are presently considered as normal constituents of the anogenital region. We report a case of young female, who presented with an anal papilloma. Histopathological examination revealed extensive fibrocystic changes in anogenital mammary-like glands. To date, a lot of benign changes and a wide range of benign and malignant neoplasms have been reported in these glands. However, extensive fibrocystic change of these glands in anal region is very rare. In addition, fibrocystic disease of anal mammary glands, masquerading clinically as an anal papilloma, has not been reported in literature. Hence, it is essential for clinicians and the pathologists to be aware of such a rare presentation. The features of fibrocystic disease in perianal region are also discussed.

## 1. Introduction

In anogenital region, mammary type glands constitute a normal component and are referred to as anogenital mammary-like glands (AGMLG) [1, 2]. These glands share several features in common with normal breast tissue like expression of estrogen and progesterone receptors [3]. Nonneoplastic lesions, benign, and malignant neoplasm arising in AGMLG are also similar to those arising in the breast. We report a case of young female, who presented with an anal papilloma on clinical examination, and the final histopathological evaluation revealed extensive fibrocystic changes (FCC) in the AGMLG.

## 2. Case Report

**Figure 1:**
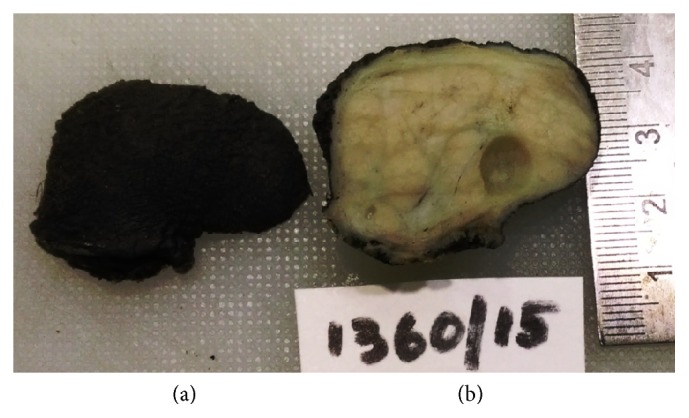
Gross appearance. (a) Smooth external surface of the polyp. (b) Cut surface is gray white, firm, and lobulated with cystic spaces.

**Figure 2:**
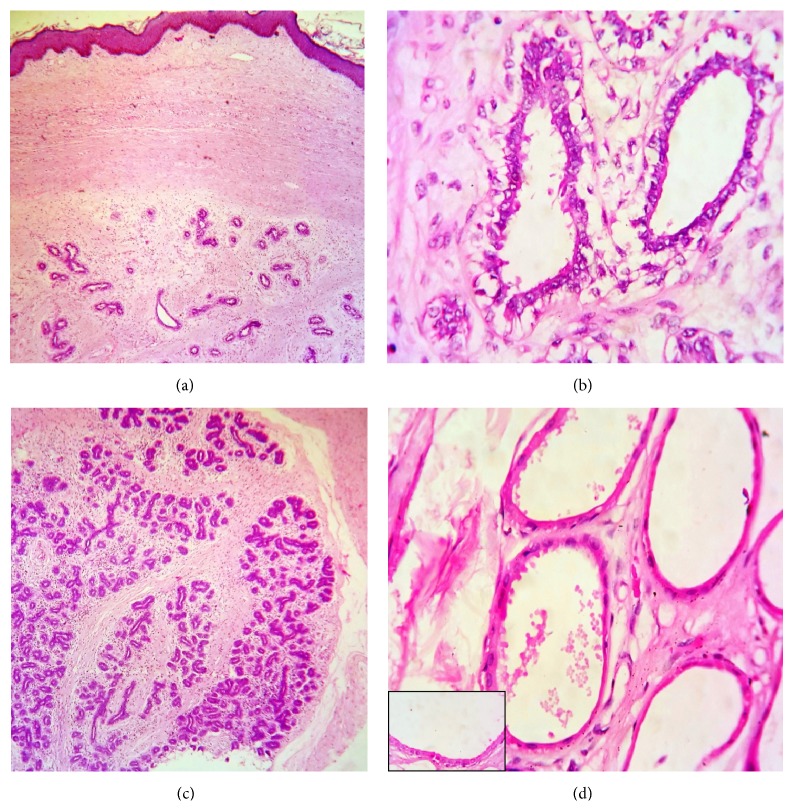
Microscopic appearance. (a) Anal skin with subepithelial tissue showing terminal duct lobular units. H&E 40x. (b) Ducts lined by bilayered epithelium. H&E 100x. (c) Areas of adenosis. H&E 40x. (d) Areas of cystic change. H&E 100x. (Inset, apocrine change with apical snouts, H&E 400x).

A 26-year-old female presented to the surgical outpatient department of our hospital with complaints of swelling in the anal region of one-year duration. Anal examination revealed a firm mobile mass at 3 o'clock position. Perrectal examination was normal and the clinical diagnosis was that of an anal papilloma. Excision biopsy of the papilloma was performed and the specimen was received in our pathology department in 10% neutral buffered formalin. Gross examination revealed a skin covered polypoidal mass measuring 3.5 × 2.5 cm. External surface was smooth (Figure 1(a)). Cut surface was gray white, firm, and lobulated with a focal tiny cyst measuring 0.5 cm in greatest dimension (Figure 1(b)). Routine histopathological examination of hematoxylin and eosin stained slides revealed skin with unremarkable epidermis and subepithelial tissue showing mammary-like glands composed of terminal duct lobular units (Figure 2(a)). The ducts were lined by bilayered epithelium, inner cuboidal epithelium, and outer myoepithelial cells (Figure 2(b)) with many of them exhibiting features of fibrocystic disease like adenosis (Figure 2(c)), cystic change (Figure 2(d)), apocrine metaplasia showing abundant eosinophilic cytoplasm with apical snouts (Figure 2(d), inset), and fibrosis in stroma.

## 3. Discussion

Ectopic breast tissue (EBT) may be present at any site along the primitive embryonic milk lines, extending from the axillary to the inguinal region [4]. Mammary type glands were reported in vulva by Hartung in 1872; these were for long considered as EBT representing the caudal remnants of the milk ridge [2]. But EBT can also be seen in anogenital regions or other unusual sites such as eyelid, nasal area, prostate gland, and gallbladder [5], which cannot be explained by the primitive milk line theory. Van der Putte proposed that, in the anogenital region, these mammary-like glands constitute a normal component [1, 2] and these glands are, at present, referred to as anogenital mammary-like glands (AGMLG).

The histology of these glands ranges from complex branching lobular units like those of normal breast to simple glandular structures surrounded by a loosely or densely fibrotic stroma [1]. Excretory system in these glands is also varied and the ducts have a luminal columnar cell lining exhibiting apocrine secretion and basal myoepithelial cell lining. Just before entering the epidermis, the ductal lining becomes stratified squamous epithelium and the myoepithelial cell layer is lost. Occasionally, small clear cells called Toker cells are present singly or in small nests surrounding the openings of these glands. These glands have mixed apocrine, eccrine, and mammary gland histology [2].

Nonneoplastic and neoplastic lesions occurring in AGMLG are similar to those arising in the breast [6]. A wide range of neoplasms can arise in these AGMLG including adenoma of lactating type, hidradenoma and syringocystadenoma papilliferum, fibroadenoma, phyllodes tumor, pseudoangiomatous stromal hyperplasia, extramammary Paget's disease, and other malignancies similar to those which arise in the breast [7]. These glands can also show nonspecific epithelial and stromal changes; a few of these are similar to the changes in the benign mammary diseases including lactation-like changes, columnar cell hyperplasia, columnar cell change, features like that of sclerosing adenosis, usual duct hyperplasia, atypical duct epithelial hyperplasia, flat epithelial atypia, satellitosis, fibrocystic change, epithelial-myoepithelial hyperplasia, lamprocyte-like changes, various metaplastic changes affecting epithelium like apocrine, oxyphilic, clear cell or squamous metaplasia, and stromal elastosis. Many of these changes do not have a clinical importance and however may lead to potential diagnostic pitfalls and hence clinicians and pathologists should be aware of such possibilities [7].

Fibrocystic change in the breast also referred to by many terms such as fibrocystic disease (FCD), fibrous or fibrocystic mastopathy, fibroadenosis cystic, and mammary dysplasia is a benign alteration in mammary tissue consisting of cystic change of the terminal duct lobular unit, with or without associated stromal fibrosis [8]. FCC is usually considered as an exaggerated physiologic response rather than a disease [9]. Microscopic features of FCC include cystically dilated round to oval spaces lined by a single or double layer of epithelial cells and attenuated myoepithelial cells: apocrine metaplasia in which the lining cells exhibit columnar morphology with abundant eosinophilic granular cytoplasm, uniform round to oval nuclei, and luminal cytoplasmic projections referred to as apical snouts: intralobular stroma showing prominent fibrosis and sclerotic changes: rupture of the cysts accompanied by secondary inflammatory reaction: microcalcifications which can be seen within the cyst lumen or in the surrounding connective tissue stroma; a mild degree of adenosis or microscopic expansion of the lobules by an increase in the number of acinar structures in the lobule that can be present [10, 11].

Anogenital mammary-like glands can undergo changes analogous to that of FCD of the breast including cystic change, periductal fibrosis, and apocrine metaplasia. FCD in AGMLG can have a wide range of microscopic features depending upon the predominant element of the disease [9]. However, the crucial histopathological alterations include cystic change, fibrosis, apocrine metaplasia, calcification, epithelial hyperplasia, and chronic inflammatory infiltrates. Similar to that of the mammary tissue, FCD-like changes can be seen focally in other lesions arising in the anogenital mammary-like glands [9].

## 4. Conclusion

To conclude, we report a case of fibrocystic change in anogenital mammary-like glands presenting as an anal papilloma. These clinically innocuous changes may however lead to potential diagnostic pitfalls and hence clinicians and pathologists should be aware of such possibilities.
